# Multinational corporations and infectious disease: Embracing human rights management techniques

**DOI:** 10.1186/2049-9957-3-39

**Published:** 2014-11-03

**Authors:** Kendyl Salcito, Burton H Singer, Mitchell G Weiss, Mirko S Winkler, Gary R Krieger, Mark Wielga, Jürg Utzinger

**Affiliations:** Department of Epidemiology and Public Health, Swiss Tropical and Public Health Institute, P.O. Box, CH-4002, Basel, Switzerland; University of Basel, P.O. Box, CH-4003, Basel, Switzerland; NomoGaia, 1900 Wazee Street, Suite 303, Denver, CO 80202 USA; NewFields, LLC, Denver, CO 80202 USA; Emerging Pathogens Institute, University of Florida, Gainesville, FL 32610 USA

**Keywords:** Infectious diseases, Human rights, Systems-based interventions, Multinational corporations, Corporate social responsibility

## Abstract

**Background:**

Global health institutions have called for governments, international organisations and health practitioners to employ a human rights-based approach to infectious diseases. The motivation for a human rights approach is clear: poverty and inequality create conditions for infectious diseases to thrive, and the diseases, in turn, interact with social-ecological systems to promulgate poverty, inequity and indignity. Governments and intergovernmental organisations should be concerned with the control and elimination of these diseases, as widespread infections delay economic growth and contribute to higher healthcare costs and slower processes for realising universal human rights. These social determinants and economic outcomes associated with infectious diseases should interest multinational companies, partly because they have bearing on corporate productivity and, increasingly, because new global norms impose on companies a responsibility to respect human rights, including the right to health.

**Methods:**

We reviewed historical and recent developments at the interface of infectious diseases, human rights and multinational corporations. Our investigation was supplemented with field-level insights at corporate capital projects that were developed in areas of high endemicity of infectious diseases, which embraced rights-based disease control strategies.

**Results:**

Experience and literature provide a longstanding business case and an emerging social responsibility case for corporations to apply a human rights approach to health programmes at global operations. Indeed, in an increasingly globalised and interconnected world, multinational corporations have an interest, and an important role to play, in advancing rights-based control strategies for infectious diseases.

**Conclusions:**

There are new opportunities for governments and international health agencies to enlist corporate business actors in disease control and elimination strategies. Guidance offered by the United Nations in 2011 that is widely embraced by companies, governments and civil society provides a roadmap for engaging business enterprises in rights-based disease management strategies to mitigate disease transmission rates and improve human welfare outcomes.

**Electronic supplementary material:**

The online version of this article (doi:10.1186/2049-9957-3-39) contains supplementary material, which is available to authorized users.

## Multilingual abstracts

Please see Additional file 1 for translations of the abstract into the six official working languages of the United Nations.

## Background

Infectious diseases have been closely linked with business interests throughout history. The spread of infectious diseases along trade routes facilitated the proliferation of plague in Europe in the 1300s and various other epidemics in the ensuing centuries, disrupting social interactions and commerce [1]. With the industrialisation of the shipping industry at the turn of the 20^th^ century, jobs, communication, wealth, goods and infectious diseases spread through ports with renewed force [2, 3]. Trade through New York City’s port brought in more than half of the national federal budget, but it also brought typhus, yellow fever and cholera epidemics to the United States of America in the 1890s [4]. Through the port, the economy grew, while infectious diseases spread through slums and immigrant enclaves, striking the poor hardest with harsh, socially and economically debilitating quarantines [4]. Then – as now – the plight of those affected by disease was not merely physical ill-health, but the social, economic, political and environmental disempowerment that did – and still today does accompany illness.

A cadre of modern-day “infectious diseases of poverty” has been identified, which primarily persist in low-income and middle-income countries, where foreign investment is growing the fastest. They include the infectious diseases mentioned above, as well as malaria, tuberculosis, HIV/AIDS and many other vector-borne, bacterial, helminthic and viral diseases [5, 6]. High rates of infectious disease and polyparisitism are well documented as both an indicator and a promulgator of poverty. Although the wealthy can also be affected by them, these diseases thrive in conditions of scarcity – of food, shelter, clean water, improved sanitation, income and education – and trap populations in continued, entrenched poverty [7–9]. In many cases, this entrenchment is compounded by corruption and failures of governance. Companies can be complicit in the spread of these diseases, but they can also be powerful players in controlling them.

### Infectious diseases and human rights

#### A governance framework

Infectious diseases can be understood through a human rights framework, when the framework is properly and effectively applied. The economic dimensions of disease, associated with conditions of scarcity mentioned above, alongside social-ecological systems, are analysed in human rights terms under the umbrella of economic, social and cultural rights. Meanwhile, the institutional dimensions of disease spread, such as corruption, health system failures, political weakness and institutional ineptitude, colonise the space of civil and political rights [10]. The human rights framework is intended to strengthen the relationship between human health and human dignity, as experienced through protections and entitlements, codified in international declarations and instruments.

These instruments – referred to collectively as the International Bill of Human Rights – also depend upon “duty bearers” meeting their allocated responsibilities [11]. Duty bearers are entities charged with ensuring that all rightsholders enjoy these rights. In this capacity they try to remediate the conditions that result in the entrenched, vicious cycle of diseases and poverty from which rightsholders suffer.

The power to spread infectious diseases where they are prevalent, and the power to prevent them, is held by the bodies controlling socioeconomic, environmental and political contexts: governments, intergovernmental organisations and business enterprises. Governments have historically been designated primary duty bearers, though they have not always succeeded in fulfilling their duties [12, 13]. Recognising that some states lack the capacity – or will – to fulfil the right to health, the International Bill of Human Rights accords an additional responsibility to other state parties, through “international assistance and co-operation” where a need is demonstrated [11, 14, 15]. The role of business enterprises has not, historically, been so clearly stated.

In 2011, the United Nations (UN) Human Rights Council unanimously endorsed Guiding Principles on Business and Human Rights (Guiding Principles in short). The Guiding Principles call upon companies to “respect” human rights [16]. This is not a new responsibility; “every organ of society” has been called upon to “promote respect” for human rights since 1948 [17]. However, it is a new and concrete articulation, clarifying for corporate actors the meaning of “respect” within the scope of their operations. Although human rights language is relatively new to companies, it has gained traction. Roughly half of the world’s largest public multinational corporations have embraced some dimension of human rights responsibility, many in response to the Guiding Principles. The major petroleum and mining associations have developed human rights stances supporting the Guiding Principles, and the Food and Agriculture Organization (FAO) has made the language of the Guiding Principles central to good practice on agriculture projects [18–20]. The corporate acceptance of human rights responsibilities is on the rise, documented through the proliferation of human rights policies and the growing demand for human rights reporting [21]. A step in fulfilling the responsibility to respect human rights is the conduct of “human rights due diligence,” which ensures that companies know how their operations may affect the lives of their workforce and surrounding communities, through environmental and social impacts, health effects, economic shifts, political affiliations and labour rights. The designated scope of corporate responsibility with regard to diseases is markedly more limited than that of government, formally restricted only to areas where companies have impacts. Yet the actions of companies should not be isolated from the initiatives of global public health practitioners, and in practice companies often do far more than host states with regard to public health [22, 23].

This paper proposes a method for broadening multinational corporations’ efforts to control, monitor and eliminate infectious diseases where they affect societies and businesses, using the Guiding Principles human rights framework. First, it presents the relationship between human rights and infectious diseases of poverty. Next, it examines corporations as human rights “duty bearers” where they operate, identifying the potential impacts they have on the spread of disease and the various ways infectious diseases affect their business interests. As an outlook, our piece proposes an approach for integrating business enterprises into ongoing initiatives for preventing, controlling, monitoring and eliminating infectious diseases, using systems-based approaches that holistically examine the conditions that promote disease spread. This approach benefits from the backing of the business community’s support of the UN Guiding Principles [16].

### An analytical framework

Outside of the corporate realm, health practitioners have struggled to convert the aspirational ideals of human rights into actionable tools and outcomes [24]. Instead, the human rights failings of states have acted as barriers to interventions. Good governance – codified in human rights instruments as the “right to public participation” – and access to affordable, quality, culturally appropriate healthcare – codified as the “right to health” – are vital for many successful disease control interventions. “Security of person”, meaning freedom from violence, and an informed and engaged public (which is achieved by educating citizens in line with the “right to education”) help empower people to seek treatment, or at the very least to attend school where treatment is often provided. Sometimes environmental conditions must be targeted where disease vectors persist, in order to reduce reinfection (as for onchocerciasis control activities in Africa) [25, 26]. These environmental interventions are a process of promoting the “right to a clean and hygienic environment.” However, a mixture of factors including budgetary limitations, ineptitude or state-driven conflict can create a milieu in which the achievement of both human rights protections and positive human health outcomes is inhibited. Health practitioners are rarely positioned to unilaterally affect change in these arenas.

Multi-pronged, integrated, intersectoral programmes have generated palpable public health gains in several interventions, as for integrated management of childhood illness (IMCI) [27, 28]. Where integrated health programmes have been augmented with the human rights framework, additional value may be added. For example, the establishment of technical guidance on human rights-based approaches to maternal and child care has enabled health practitioners to address systemic governmental and international failures that lead to negative human rights outcomes, while also identifying structural conditions that disempower women, politically, socially and economically [29].

**Figure 1:**
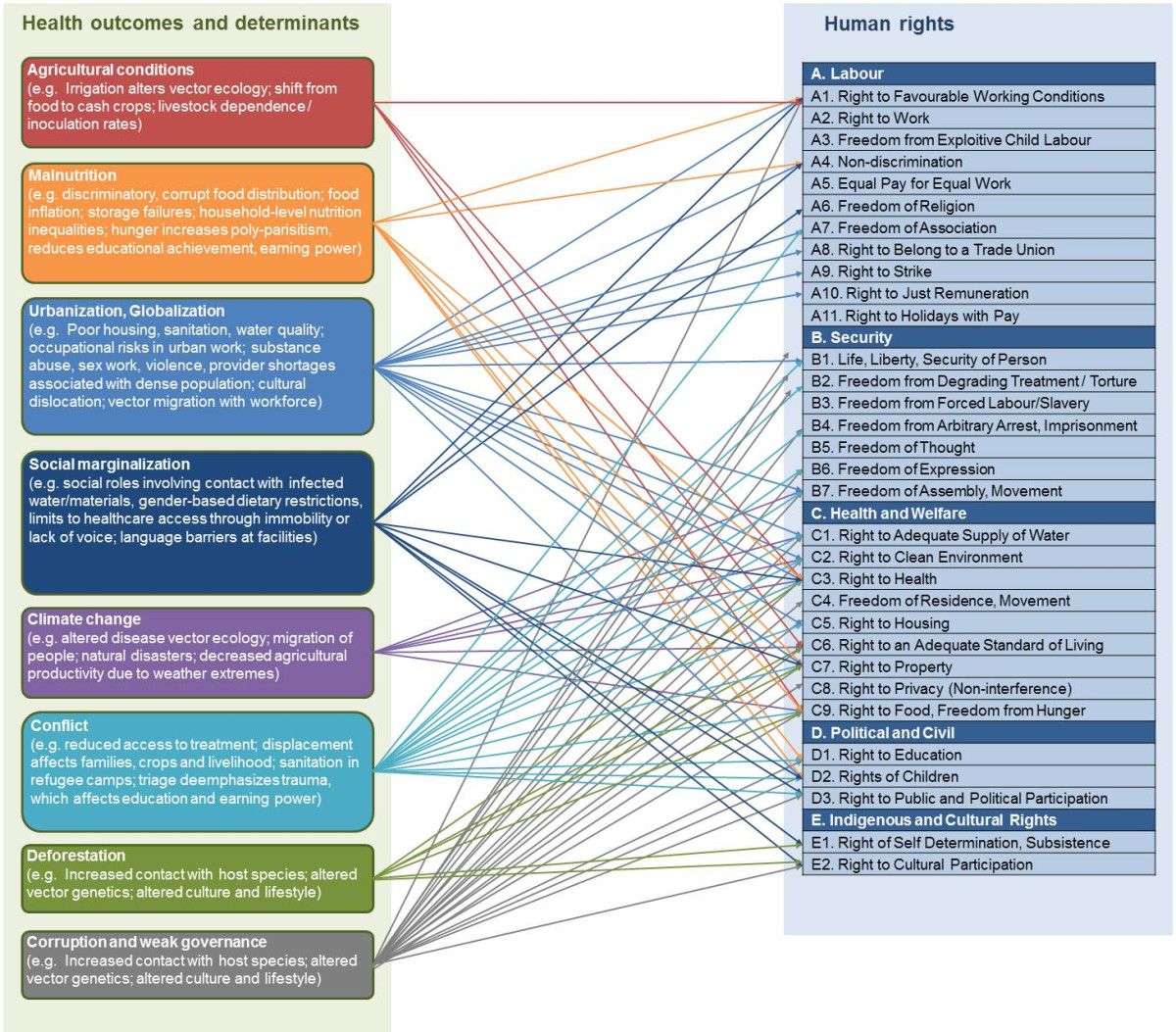
**Linkages between determinants of health and human rights affected by infectious diseases of poverty.**

Figure 1 connects socioeconomic, cultural and political conditions to the relevant human rights affected, demonstrating the intimate connections between both the human rights and health outcomes resulting from external forces. The column labelled “Outcomes of ill-health” is drawn directly from the World Health Organization (WHO) technical guidance and supplemented with a key consideration recognised in the literature on neglected tropical diseases: corruption and governance failures [6]. The column labelled “Relevant human rights affected” was constructed through a Delphi method, deriving rights from the International Bill of Human Rights [11].

Infectious diseases are a measurable outcome of, and a contributor to, a wide variety of unrealised and unprotected human rights, as visualised in Figure 1 and thoroughly reported elsewhere. Illness affects social engagement (“right to public participation”), academic performance (“right to education”) [30–32], long-term earning power (“right to an adequate standard of living”) [12, 33, 34] and, for HIV and other highly stigmatising diseases, personal safety (“the right to security of person”) [35–37].

Additionally, negative human rights conditions perpetuate infectious disease spread through failures of governance [38, 39]. Government corruption can reduce available resources for public health initiatives. Widespread graft can press international donors to withdraw aid, further reducing resources for achieving the highest attainable standard of health for citizens. Where logistics, corruption and supply chain management result in socioeconomic disparities in coverage, the right to public participation is violated alongside the right to health, even if the inequitable distribution of coverage is inadvertent [40]. The “right to public participation and equal access to public service” can be violated by the syphoning of funds from public coffers. In conflict settings, governments can contribute to conditions of insecurity, militarising transportation routes or limiting access to treatment for certain sub-populations. The role of state security forces as they interact with existing social fissures and resource disparities can make access to treatment impossible, as has been the case in Nigeria, South Sudan, Democratic Republic of the Congo and, most recently, Syria [12, 13, 41, 42].

Within the scope of the right to health, infectious diseases have compounding effects. For example, an infection might weaken immune responses and lower nutritive intake, resulting in higher morbidity from a variety of communicable and non-communicable diseases (NCDs) [43]. Affected sub-populations have lower access to health knowledge, treatment and services, which heightens the risk of co-infection with other infectious diseases of poverty [44]. Furthermore, the spread of infectious diseases is multiplicative as transmission rates rise [45, 46].

## Methods

Ethical clearance was sought from the ethics commission of Basel Stadt, where the Swiss Tropical and Public Health Institute is located (Ethikkommission beider Basel reference number 304/13), as well as the National Research Council of Malawi, via the National Health Sciences Research Committee (NHSRC Reference number 1215).

### Past efforts and present duties: multinational corporations as duty bearers

The role of companies, in terms of both health concerns and human rights concerns, differs from that of global health agencies in obvious and crucial ways. For health agencies, the promotion of global health is central to their mission, and human rights is an advocacy argument, reminding parties of their commitments to strive for the highest attainable standards of care for all individuals, regardless of race, gender, religion, socioeconomic status or other marginalising characteristic [47]. For businesses, health and human rights have had an evolving role in decision-making, and neither is usually considered central to business operations. As such, a clear delineation of the corporate duty to respect human rights is useful in a discussion of corporate involvement combatting infectious diseases.

Companies have been investing in infectious disease interventions for centuries, because the productivity gains associated with reducing transmission proximal to where they were operating outweighed the cost of control measures. However, the cost analysis has not always worked out to favour human rights. In one of the United States of America’s greatest industrial health disasters, employers of the Gauley Bridge construction site exposed thousands of workers to silica dust, resulting in over 1,500 deaths from silicosis, pneumonia and tuberculosis, none of which the implementing company, Rinehart & Denis, or its contracting company, Union Carbide, prevented or treated [48].

### Past efforts: the business case

Laggards like Rinehart & Dennis persist today but they are not the focus of this paper, because they are not the companies that set trends for the future. Instead, we are interested in the growing number of companies that are aiming to do better. Some are acting in so-called enlightened self-interest, finding a profit motive for doing good. Others state an intention to explicitly benefit public welfare through their operations. Corporate motives are difficult to identify, but the outcomes of their actions can be evaluated to establish best practices for the future. This is important, as the globalisation of business is on the rise.

With roughly 80,000 multinational corporations averaging 10 foreign affiliates, multinational companies generate approximately 11% of global gross domestic product (GDP) [49]. Today’s corporate impacts on global systems are historically unprecedented, associated with large-scale agriculture, land clearance, urban expansion and industrialisation [50]. Companies developing infrastructure-intensive operations where infectious diseases are widespread can exacerbate transmission simply through their core business operations – building dams and transportation corridors, hiring and moving around construction teams, housing workers and other activities. Yet, the public health challenges companies face, and the remedies they pursue in the process of global expansion, have a long history.

Corporate actors operating in the tropics were early contributors to public health, spending millions in recognition that a healthy workforce was a productive one. Multinational mining, engineering and agribusiness firms instituted environmental management programmes to control malaria, yellow fever and other infectious diseases near their operations throughout the early 1900s, sometimes decades before government public health programmes caught up in Latin America, the Middle East, Asia and Africa [51–53]. In one example, Firestone Plantations Company conducted extended surveys and treatment of populations affected by human African trypanosomiasis in Liberia during the 1940s. The company collaborated with the WHO and the national government in a mass-treatment programme to eliminate yaws between 1957 and 1959, simultaneous with a control programme targeting smallpox, and assisted the WHO to conduct a pulmonary tuberculosis survey in 1962.

With the biomedical surge of the 1960s, pharmaceutical companies became partners with non-governmental organisations (NGOs), governments and extractive industries to control and eliminate lymphatic filariasis, onchocerciasis, trachoma, malaria and HIV/AIDS [53]. Some partners have profited from these interventions, some have taken on significant expense and some may have balanced the two [25, 54–56]. Merck’s Mectizan Donation Programme to treat and prevent onchocerciasis may have fit each of these descriptions over its 27 years of operation. Ivermectin was and is one of the firm’s most profitable drugs, used on livestock and pets to control heartworm. When Merck discovered its human utility, it sought buyers but found none, so it offered to donate the drug (under the name Mectizan) indefinitely to any country that could not afford it. By 2004, the programme had cost Merck over US$ 200 million, but in exchange, the company received tax write-offs, positive press and the commitment of partner organisations to prevent human-directed treatments from being administered to animals, which would undermine veterinary profits [57, 58]. In another example, in managing HIV/AIDS in sub-Saharan Africa, one mining company estimated that at its peak, the epidemic would add 8-17% to payroll costs, and another began training two to three workers for a single job, assuming at least one would die of AIDS [59]. To control the costs of lost labour, and perhaps also to support public welfare, mining companies intensified their investment in public health, partnering with a variety of organisations to provide health personnel with strategic access to working populations, and provide workers with access to treatment [60]. Over time, these initiatives have broadened to address comorbidities with tuberculosis and other illnesses [61].

The collaborative approaches, across industries, aimed at tackling various infectious diseases in tandem, have led the way to increasingly holistic approaches to disease control, accounting for the broader socioeconomic and political conditions that affect projects and worker welfare. Where such broader contexts have been ignored, results have been mixed. As the Firestone Plantation Company learned over decades in Liberia, public health is not the only contextual concern that can affect productivity, nor can public health be addressed strictly through health interventions. Worker welfare proved itself to be more comprehensive than the absence of illness as early as 1949 when Firestone workers first went on a wage strike. By the time the company’s infectious disease programmes were firmly established and the workforce (and dependents) had achieved near universal health care access, workers had begun recognising labour issues beyond the inadequate housing that fostered disease spread [62]. A 1963 strike of 20,000 Firestone workers shut down all 45 divisions of the plantation’s operations. Workers demanded higher wages, improved housing, shorter working hours and better work conditions – essential human rights in a context where wages were insufficient to buy rice, housing had been unrepaired for decades and workdays reached 14 hours [63–65]. Labour disputes persisted until Liberia’s civil war and beyond. In 2005 the company, by then owned by Bridgestone, faced an Alien Tort Claims lawsuit filed by the workforce against Firestone’s use of forced labour, child labour, cruel and unjust treatment and negligent supervision creating an unsafe workplace [66]. Also by then, a legal regime had been established in Liberia to protect workers’ rights.

The corporate-government agreements managing social, environmental and health impacts were initially specific and voluntarily negotiated. Many have become generalised and gained the force of law. Since the 1970s, through the passage of national environmental protection acts, companies have been required to mitigate their impacts on the human and natural environment when their activities are likely to cause harm [67]. Though in early decades analysis of the “human environment” was often minimised, both stakeholder pressure (particularly on multi-lateral funding agencies such as the World Bank’s private sector lending arm, the International Finance Corporation, the Asian Development Bank and the European Bank for Reconstruction and Development) and overt legal challenges (in the United States of America) gave a substantial boost to the field of health impact assessment (HIA) in the 1990s, specifically to fill the “health” gaps in environmental and social impact assessments [68].

There are two main lessons to be drawn from Firestone’s experience. First, corporate impacts on communities affect corporate revenues. Land rights, labour rights, civil rights and social and environmental impacts of project development can increase a project proponent’s risk of shutdowns and liabilities [69]. Second, addressing those impacts requires holistic interventions, and a good deed in one area of corporate activity does not cancel out harm elsewhere.

### Present duties: the human rights and social responsibility case

Between 2008 and 2011, acknowledgement of corporate impacts was further refined and rephrased in human rights terms, reinforcing the role of companies as “organs of society”, responsible for respecting human rights in their activities [16]. Under unanimously endorsed UN guidance, corporations are expected to identify, prevent and remediate their human rights impacts while they pursue their core business activities. The direct effect of corporate activities on transmission of infectious diseases makes it a corporate concern, because a failure to reverse those effects represents a lack of “respect” for the right to health and a number of accompanying rights affected by infection. This poses challenges for companies, but also presents an opportunity for them to adopt more effective disease management strategies and benefit from collaborations with international health agencies and national Ministries of Health (MoH). Governments and intergovernmental organisations can contribute to corporate programmes and benefit from them; the successes companies achieve within their walls or fence lines can be imparted and scaled up by governments through effective knowledge exchange and communication.

These are ideological underpinnings of the Guiding Principles, which are the current, *de facto* authority on corporate interactions with rightsholders worldwide [16]. Indeed, the Guiding Principles call on companies to respect human rights by ensuring that their operations do not violate or contribute to violations of human rights. Corporate responsibilities are also derived from the International Covenant on Economic, Social and Cultural Rights, which calls on the international community to provide technical and financial support to governments attempting to fulfil rights but lacking resources (Article 2).

**Figure 2:**
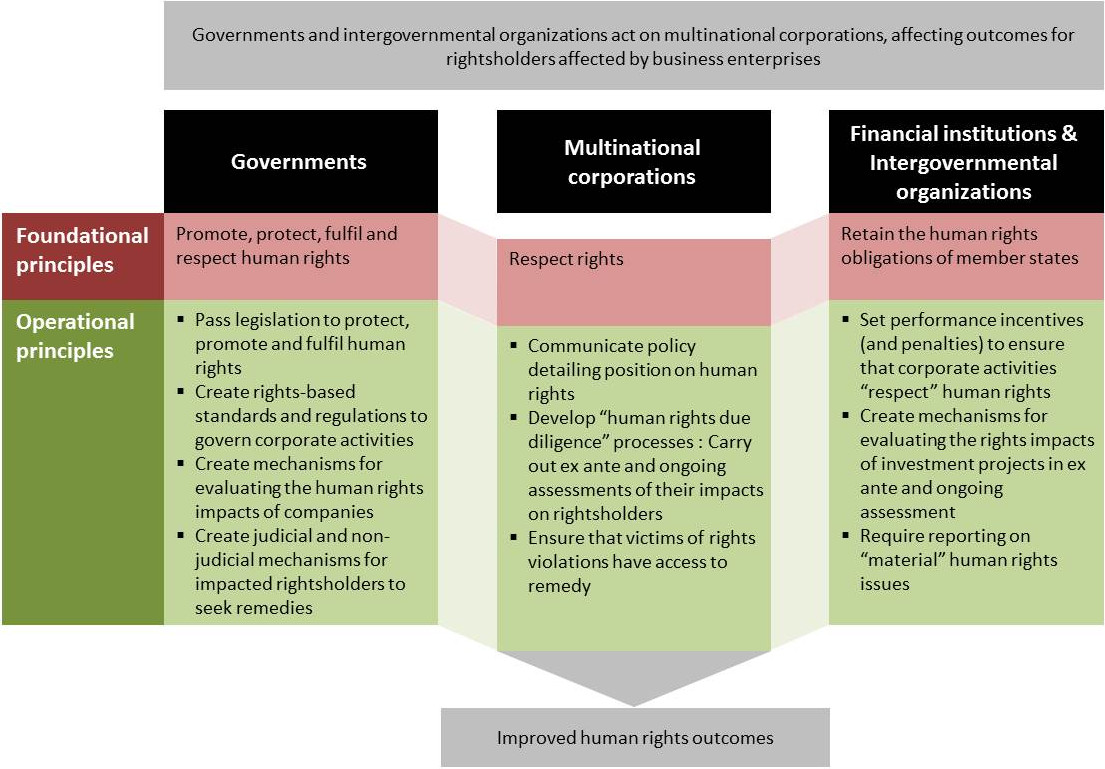
**Key players affecting human rights outcomes through infectious disease management, and their respective roles.**

The corporate impetus to holistically manage infectious diseases now has three drivers: (i) an impact prevention and remediation (or “do no harm”) principle derived from human rights responsibilities; (ii) a growing normative and legal framework; and (ii) a longstanding business case for reducing absenteeism. Companies are included in the category of international actors who are to avoid violating rights and operate in accordance with the standards of governments that attempt to protect and promote them [29, 70, 71]. What that means in practice is largely procedural: companies need to understand baseline conditions, evaluate impacts and take actions to mitigate impacts (Figure 2). Processes for evaluating human rights impacts are increasingly well-developed and in many ways linked to HIA processes [72]. Corporate activities inadvertently affect the spread of many infectious diseases, through the engineering of water storage mechanisms, the consolidation of populations in centralised areas, and the introduction of hazards that interact with infectious diseases. Dams disrupt hydrology and water-filtration processes, facilitating the spread of water-borne bacterial and parasitic diseases [73]. The assembly of construction teams and other labour forces into densely populated communities or high-capacity dormitories increases risks of communicable disease transmission [74]. Where workers relocate to a job site, they may bring endemic diseases from their home villages [50, 75]. Worksite lifestyles may increase disease spread upon workers’ return to their communities during leave, including sexually transmitted diseases, yellow fever and tuberculosis [50, 76].

Corporate projects that require the resettlement of populations living atop or adjacent to project sites have myriad and complex human rights impacts. Social dislocation can affect security of person and the rights of children. The stress of relocation often results in increased infectious disease rates, decreased educational performance by resettled children and a loss of livelihood and income as families rebuild their homes, fields and business ties. The introduction of toxins, toxicants and particulates into air is another major contributor of corporate activity to negatively impact infectious disease (and chronic disease) outcomes [77–79]. Alongside the Gauley Bridge incident mentioned above, the problematic occupational exposure history of South African mine workers to silicosis, as it fomented a national tuberculosis crisis, typifies such negative corporate human rights impacts [80].

Even the direct positive impacts of a project can result in negative health and human rights outcomes that require remediation under the “do no harm” principle. For instance, the improvement in size and reliability of food supplies, often facilitated by mechanised farming or wage labour, enables increased human and livestock population density, which increases animal-to-human and human-to-human transmission risks of infections. Likewise, as large-scale industry increases service delivery and access to a money economy to previously isolated subsistence communities, environmental impacts and economic transitions have effects on the rights to a clean environment, food, health, adequate standard of living and, for children, the right to a family life. Mechanised farming may also promote the transition to non-food crops which, on the one hand may improve access to markets and farming inputs, and on the other may affect water supply, deforestation and, over years, result in declining yields, reduced standards of living and increased presence of disease vectors [81–84].

In conflict settings, core business activities can indirectly affect disease spread, through processes and procedures that directly affect human rights. This is most apparent in situations where companies develop projects in conflict or post-conflict zones, in which even securing the project periphery can increase public insecurity, to the detriment of community welfare. One of the most thoroughly documented cases of this is the militarisation of Ogoniland in south eastern Nigeria to secure territories for Royal Dutch Shell’s oilfields. The company’s pipelines have experienced numerous breaches since operations began in 1958, resulting in degradation of farmlands and fishing grounds, which has affected nutrition in the area. Additionally, the ethnic minority Ogoni who have protested the environmental harms have been violently suppressed by ethnic majority troops from southern Nigeria. Shell’s own security personnel have not been directly linked to violence, but Shell imported weapons for the Nigerian military [85, 86]. The insecurity and dislocation have had wide-reaching public health effects [87]. In another example, corporate security forces protecting mines in Sierra Leone contributed to atrocities during the civil war. The violence has been tied to myriad lingering negative health impacts [88].

## Results and Discussion: implications on the ground

### Limitations of a human rights framework without enforcement capabilities

A human rights approach to operating in conflict settings has nominally been applied by many extractive companies, through their participation on the Voluntary Principles on Security and Human Rights. This mechanism has embodied two of the major drawbacks of the human rights framework, namely (i) that it is voluntary and that (ii) it prioritises certain rights to the exclusion of others.

Without express, contractual or legal advances, the human rights regime is seen by some as “toothless” [89]. Companies are not well acquainted with the human rights framework and, with little guidance, many have ignored it. Stiglitz and others refer to this current system as “global governance without global government” [90, 91], whereby a framework exists for sharing duties, but no implementing agency can ensure that each duty bearer plays its part. Companies in the past have tended to pick and choose among the rights they deem relevant [92].

However, cherry-picking rights poses risks. The confluence of human rights duties and infectious disease management is convenient but also complicated. Implemented partially or improperly, the human rights approach can be ineffective, or at worst, counterproductive [47]. One arena where the human rights approach has garnered legitimate criticism in the public health (and private business) sphere is in the HIV/AIDS pandemic. For migrant workers seeking private sector jobs in Oman, for example, a negative HIV test certificate is required for entry, to the detriment of the right to privacy, work, non-discrimination and security of person [93]. Conversely, the public health sector’s focus on reversing stigma and protecting privacy rights became a factor in the global spread of HIV. Vital and exacting standards for protecting the seropositive from stigma, discrimination and the psychologically damaging effects of a positive diagnosis of a then-untreatable disease did much to protect vulnerable groups when medicine had little to offer HIV/AIDS patients. However, as treatment improved, destigmatisation progressed (though, notably, not for all at-risk groups, such as homosexual males in Africa), and the privacy standards remained, while the human rights risks of not knowing one’s HIV status began proliferating. HIV has converted into a complex chronic illness requiring comprehensive long-term management, but management is hindered by the very privacy standards that offered the seropositive their greatest initial protection [94]. In sub-Saharan Africa, the result has been sweeping impacts on socioeconomic rights for families impoverished by illness and fragmented by death [95, 96].

A narrow focus on a single rightsholder group has been similarly problematic. Perhaps the most historically powerful example of this is embodied in the “environmental justice” movement in the United States of America, which chronicled the systematic disregard for the health of minority populations living in proximity to industrial sites at the same time that occupational health and safety regulations were ensuring that (non-minority) employees were better protected from those same hazards [97, 98]. The result of this racially-based disregard was a series of lawsuits culminating in a legal movement and a (far from complete or perfect) new global sensitivity. It is because the human rights lens takes the long- and short-term, direct and indirect, and single and cumulative impacts into consideration that it offers value. Neglect of either particular rights or particular rightsholders poses problems. As such, the full suite of rights and rightsholders should be considered systematically.

### Blending corporate “do no harm” with state “duty to protect”: the state role in the guiding principles

Just as the human rights framework weakens when implemented for particular rights or rightsholders, it also loses effectiveness when implemented to exclude certain duty bearers. Again, the Guiding Principles provide direction to integrate inter-governmental organisations, government bodies and business enterprises in the protection of human rights, systematically and holistically. They lay out a system of global governance incorporating the roles of governments, international financial institutions, civil society and corporations to create a network of responsible parties with interacting but not overlapping duties. Examples above generally present states as useful partners with limited means, or as barriers to change. They can do more. Fox and Meier (2009) have proposed that states could pass laws codifying the duties of international financial players to include respect for human rights [99]. The Guiding Principles, too, instruct states to “consider the full range of permissible preventative and remedial measures, including policies, legislation, regulations and adjudication” [16]. Within the scope of direct foreign investment, some states have already begun doing this. In 2013 the Government of Honduras signed a Memorandum of Understanding (MoU) with BG International, a hydrocarbon exploration and extraction company, incorporating respect for human rights as a core commitment of the partnership. The MoU was published, temporarily, through the Extractive Industries Transparency Initiative (EITI), potentially providing guidance for other states and extractive companies.

To be fully effective, such laws, contracts and regulations should conform to the criteria for “respect” that include the active duty of investigating impacts. First, companies should have a policy detailing their position on human rights for all rightsholders affected by operations, including workers and neighbouring communities. Second, they should develop “human rights due diligence” processes, documenting the steps they have taken to ensure that their activities do not violate or contribute to the violation of human rights. Finally, they should develop mechanisms, complementary to those of states, to ensure that victims of rights violations have access to remedy. By requiring these actions of companies, and evaluating the outputs produced by companies, governments can increase their understanding of corporate impacts, understand the epidemiological implications, and collaborate with companies to find solutions [16].

The comprehensive human rights approach has advantages over direct approaches to health, or even the right to health, as past efforts to target health directly have been limited by the assumption that health belongs within the scope of medicine, subject to the budget limitations of the MoH [99]. A human rights approach, which incorporates the full suite of rights, recognises the interrelationship between health and social determinants of health, requiring parties to address the non-linear relationships between impacts and outcomes. Private sector health and infectious diseases initiatives deserve praise for their successes [23, 100], but cautionary tales from rights-neglectful initiatives like Firestone’s should help steer companies towards holistic and rights-respectful approaches.

### A role for international organisations within the guiding principles

The Guiding Principles also call for greater policy coherence at the international level, setting out a role for intergovernmental institutions that aligns with the human rights obligations of their member states. For the WHO, international financial institutions and trade associations, these obligations are the foundational human rights instruments, to which all or most states are members. The strong and broad support that the Guiding Principles enjoy empower policymakers to implement their recommendations, including adopting processes to ensure that corporate activities “respect” human rights and intergovernmental institutions find smart ways to collaborate with companies that are already on the ground in areas to address endemic diseases simultaneously with longstanding poverty.

Delving deeper into “human rights due diligence,” companies are expected to carry out *ex ante* and ongoing assessments of their impacts on rightsholders. MoH, in collaboration with the WHO, the Global Fund to Fight AIDS, Tuberculosis and Malaria, the Centers for Disease Control and Prevention (CDC) and other health agencies keep records of epidemics, incidence rates and prevalence rates, which comprises baseline conditions for companies investing in new projects in these locations. These data might be of low quality or reliability, but they can allow skilled and experienced assessors to make qualitative conclusions about risks. Leading companies are already commissioning human rights impact assessments (HRIA), which – when they are done properly – analyse such data. At a Uranium mine in northern Malawi owned by an Australian company, Paladin Energy, the initial paucity of local data prompted the company to begin tracking HIV testing, treatment and counselling and bolstering the Malawian government’s statistics. ExxonMobil is currently running a much broader infectious disease monitoring programme at its operations in Papua New Guinea, using improved national data to track changes in the project area and to design interventions. Such alliances can be costly in some cases but have proven effective [22, 101]. Although ExxonMobil is not currently using its health findings to inform its human rights approach, Paladin is. The tracking Paladin conducted at its Malawi mine enabled the company to benchmark access to treatment in the project compared to the rest of the nation. The most recent human rights monitoring report revealed that Paladin’s programmes insulated local communities from a national antiretroviral treatment stock-out, positively impacting the right to health while the government was unable to fulfil its duty. A dynamic and iterative approach to understanding the causes and outcomes of health interventions will enable all duty bearers to tailor interventions to local conditions.

## Conclusions

The impacts of infrastructure projects differ across regions, contexts and industries [102–104]. For this reason, the human rights approach considers the direct and indirect interactions between a corporate project and its operating context. This holistic understanding not only enables companies to identify and manage risks, but to maximise positive impacts.

Vertical, disease-specific interventions do not suffice to protect business interests or human rights, partly because they cannot pre-emptively disrupt the cycle of disease and poverty that characterises infectious diseases [105]. A human rights approach examines the full suite of interconnected rights as it applies to the full range of rightsholders and duty bearers. The human rights lens identifies the risks and their associated appropriate remediation measures as well as the sweeping positive impacts that must also be considered in project development. Major petroleum companies have recognised the value of comprehensive, holistic interventions.

The very clear relationship between occupational illnesses, chronic diseases and infectious diseases necessitate that they be tackled together through a holistic approach [106, 107]. Zoonotic diseases, too, rest under this umbrella, with the OneHealth strategy already presenting a model for integrating the economic, social and health drivers and outcomes of holistic interventions [108]. Although this paper focuses on infectious diseases, leading health initiatives have already begun expanding the health lens to include NCDs and chronic illnesses that can result as much from the benefits and risks of globalisation [109, 110]. There is a growing recognition that increased standards of living and availability of processed foods and beverages at locations where globalised business changes local diets is affecting coronary heart disease rates, diabetes myelitis and complications of obesity [111].

Fortunately, many corporate impacts are inherently positive and promote a “virtuous cycle”. Improving education, nutrition, knowledge and empowerment creates positive feedback loops that can neutralise or reverse the cycle of illness and disempowerment that are characteristic for infectious diseases of poverty. These inputs are credited with much of the improvement in public health and life expectancy in Europe since the end of World War II [112, 113]. In our interconnected world, research priorities are shared across industries and disciplinary fields [114]. In part because corporate investment in communities often includes contributions to education, nutrition, equality and access to information, some companies have seen striking success in their public health interventions. In the Amazon, forest clearance is correlated to elevated malaria incidence, with the exception of corporate-sponsored clearance programmes, which allocate resources to environmental controls and public education campaigns [115]. This is a positive indication of the corporate cognisance of systems-thinking – incorporating preventive measures into activities that would otherwise pose health risks [44]. Leading companies educate communities and supply insecticide-treated nets, control vegetation and drain swamplands to reduce transmission of mosquito-borne infections and successfully manage schistosomiasis and other infectious diseases. In the course of a HRIA between 2008 and 2013, Paladin Energy identified gaps in the Government of Malawi’s HIV/AIDS prevention programme to identify treatment and control failures in their project area and fill the gap through collaborative efforts with the MoH and a variety of civil society groups [72, 116].

Through the Guiding Principles, policymakers have new tools to benefit from the presence of private sector actors in rural and resource-constrained settings, as well as a duty to ensure that these actors recognise their impacts and manage them. Systematising interventions, and integrating them into *ex ante* analyses and monitoring programmes at corporate project sites, including mines, dams, oilfields, plantations and manufacturing sites, can better protect the public health of communities and to manage financial risks to companies. Infectious diseases should be tackled together [117]. They include most zoonotic diseases that affect livelihoods and economic growth in the framework of human and animal health [118]. OneHealth interventions broaden the lens of human illness to recognise complex systemic interactions [108, 119]. Furthermore, infectious diseases considered in this analysis are one aspect in the broader context of health problems, which include environmental determinants and risk factors for NCDs. The lens for examining these complex interactions should be refined to enable consideration of the role of human rights. The human rights approach is naturally conducive to holistic analysis, and it also brings together the various duty bearers and acknowledges the diverse rightsholders affected. Corporate risk matters – projects are expensive in low-income countries, and this is where infectious diseases of poverty have their strongest hold. Companies can ensure that they are preventing negative human rights impacts while maximising workforce health and efficiency by tackling these diseases within the human rights contexts where they proliferate.

## Electronic supplementary material

Additional file 1:Multilingual abstracts in the six official working languages of the United Nations.(PDF 330 KB)

## Abbreviations

CDC: Centers for disease control and prevention
EITI: Extractive industries transparency initiative
FAO: Food and agriculture organization
GDP: Gross domestic product
HIA: Health impact assessment
HRIA: Human rights impact assessment
IMCI: Integrated management of childhood illness
MoH: Ministry of health
MoU: Memorandum of understanding
NCD: Non-communicable disease
UN: United Nations
WHO: World health organization.

## References

[1] Bos KI, Schuenemann VJ, Golding GB, Burbano HA, Waglechner N, Coombes BK, McPhee JB, DeWitte SN, Meyer M, Schmedes S, Wood J, Earn DJ, Herring DA, Bauer P, Poinar HN, Krause J (2011). A draft genome of *Yersinia pestis* from victims of the Black Death. Nature.

[2] Porter S (2009). The Great Plague.

[3] Isaäcson M (1989). Airport malaria: a review. Bull World Health Organ.

[4] Markel H (1999). Quarantine!: East European Jewish Immigrants and the New York City Epidemics of 1892.

[5] Utzinger J, Becker SL, Knopp S, Blum J, Neumayr AL, Keiser J, Hatz CF (2012). Neglected tropical diseases: diagnosis, clinical management, treatment and control. Swiss Med Wkly.

[6] Hotez P, Ottesen E, Fenwick A, Molyneux D, Pollard AJ, Finn A (2006). The neglected tropical diseases: The ancient afflictions of stigma and poverty and the prospects for their control and elimination. Hot Topics in Infection and Immunity in Children III.

[7] Ball P (2009). Poor trapped in poverty by disease. Nature News.

[8] Jha P, Mills A, Hanson K, Kumaranayake L, Conteh L, Kurowski C, Nguyen SN, Cruz VO, Ranson K, Vaz LM, Yu S, Morton O, Sachs JD (2002). Improving the health of the global poor. Science.

[9] Hotez P, Fenwick A, Savioli L, Molyneux D (2009). Rescuing the bottom billion through control of neglected tropical diseases. Lancet.

[10] Braveman P, Gruskin S (2003). Poverty, equity, human rights and health. Bull World Health Organ.

[11] Assembly UNG (1996). International Bill of Human Rights.

[12] Isah EC, Omorogbe VE, Orji O, Oyovwe L (2008). Self-reported absenteeism among hospital workers in Benin City, Nigeria. Ghana Med J.

[13] Aylward RB, Alwan A (2014). Polio in Syria. Lancet.

[14] United Nations General Assembly (1976). International covenant on civil and political rights. Resolution 220A (XXI).

[15] United Nations General Assembly (1976). International covenant on economic, social and cultural rights. Resolution 220A (XXI).

[16] Ruggie JG, United Nations High Commission on Rights (2011). Guiding Principles on Business and Human Rights: Implementing the United Nations “Protect, Respect and Remedy” Framework.

[17] United Nations. General Assembly (1948). Universal Declaration of Human Rights, vol. Resolution 217 A (III).

[18] FAO, Committee on World Food Security (2012). Voluntary Guidelines on the Responsible Governance of Tenure of Land, Fisheries and Forests in the Context of National Food Security.

[19] ICMM Secretariat (2011). Guiding principles for the implementation of the UN ‘Protect Respect and Remedy’ framework. ICMM Response to the Special Representative of the Secretary General on the Issue of Business and Human Rights.

[20] IPIECA (2012). IPIECA Participates at UN Business and Human Rights Forum in Geneva.

[21] Ruggie JG (2013). Just Business: Multinational Corporations and Human Rights.

[22] Thomason J, Hancock M (2011). PNG Mineral Boom: Harnessing the Extractive Sector to Deliver Better Health Outcomes.

[23] Whiteside A, Loewenson R (1998). Socio-economic implications of HIV/AIDS in Southern Africa. Towards a New Partnership with Africa: Challenges and Opportunities.

[24] Institute of Medicine (2012). For the public’s Health: Investing in a Healthier Future.

[25] Mackenzie CD, Homeida MM, Hopkins AD, Lawrence JC (2012). Elimination of onchocerciasis from Africa: possible?. Trends Parasitol.

[26] Prichard RK, Basanez MG, Boatin BA, McCarthy JS, Garcia HH, Yang GJ, Sripa B, Lustigman S (2012). A research agenda for helminth diseases of humans: intervention for control and elimination. PLoS Negl Trop Dis.

[27] Armstrong Schellenberg J, Bryce J, de Savigny D, Lambrechts T, Mbuya C, Mgalula L, Wilczynska K, Tanzania IM-CEHFSSG (2004). The effect of integrated management of childhood illness on observed quality of care of under-fives in rural Tanzania. Health Policy Plan.

[28] Wang L-D, Chen H-G, Guo J-G, Zeng X-J, Hong X-L, Xiong J-J, Wu X-H, Wang X-H, Wang L-Y, Xia G, Hao Y, Chin DP, Zhou X-N (2009). A strategy to control transmission of schistosoma japonicum in China. N Engl J Med.

[29] Yamin AE (2013). From ideals to tools: applying human rights to maternal health. PLoS Med.

[30] Fine PE (1982). Leprosy: the epidemiology of a slow bacterium. Epidemiol Rev.

[31] Perera M, Whitehead M, Molyneux D, Weerasooriya M, Gunatilleke G (2007). Neglected patients with a neglected disease? A qualitative study of lymphatic filariasis. PLoS Negl Trop Dis.

[32] Castro A, Farmer P (2005). Understanding and addressing AIDS-related stigma: from anthropological theory to clinical practice in Haiti. Am J Public Health.

[33] Jukes MC, Nokes CA, Alcock KJ, Lambo JK, Kihamia C, Ngorosho N, Mbise A, Lorri W, Yona E, Mwanri L, Baddeley AD, Hall A, Bundy DAP, Partnership for Child Development (2002). Heavy schistosomiasis associated with poor short-term memory and slower reaction times in Tanzanian schoolchildren. Trop Med Int Health.

[34] Weiss MG (2013). The nature of stigma and new challenges of leprosy control. 18th Annual International Leprosy Congress.

[35] Meel BL (2003). 1. The myth of child rape as a cure for HIV/AIDS in Transkei: a case report. Med Sci Law.

[36] Rohleder P (2010). ‘They don’t know how to defend themselves’: talk about disability and HIV risk in South Africa. Rehabilitation in Practice.

[37] Yeager R (2003). HIV/AIDS: implications for development and security in Sub-Saharan Africa. Civil-Military Alliance to Combat HIV & AIDS.

[38] Namuigi P, Phuanukoonnon S (2005). Barriers to measles immunization: the beliefs and attitudes of caregivers in Goroka, Eastern Highlands Province, Papua New Guinea. P N G Med J.

[39] Phuanukoonnon S, Maraga S, Gouda H, Vallely A, Horwood P, Soli K, Pulford J, Rarau P, Vengiua G, Kumai J, Cross G, Pellegrini M, Greenhill A, Siba P (2013). Report of partnership in health project (PiHP) January to June 2013. Port Moresby, PNG: Papua New Guinea Institute of Medical Research (PNGIMR).

[40] UK National Audit Office (2009). Department for International Development: Aid to Malawi.

[41] Beyrer C, Villar JC, Suwanvanichkij V, Singh S, Baral SD, Mills EJ (2007). Neglected diseases, civil conflicts, and the right to health. Lancet.

[42] Cousins S (2013). Polio outbreak in Syria. Nature Middle East.

[43] Kamal SM, El Sayed KK (2006). Immune modulation by helminthic infections: worms and viral infections. Parasite Immunol.

[44] Sachs J, Malaney P (2002). The economic and social burden of malaria. Nature.

[45] Sachs J (2009). Common Wealth: Economics for a Crowded Planet.

[46] Bleakley H (2007). Disease and development: evidence from hookworm eradication in the American South. Q J Econ.

[47] Horton R (2013). Offline: Who cares about human rights anyway?. Lancet.

[48] Cherniak M (1989). The Hawk’s Nest Incident: America’s Worst Industrial Disaster.

[49] Li S, Gaur A (2014). Financial giants and moral pygmies?: multinational corporations and human rights in emerging markets. Int J Emerg Market.

[50] Butler CD (2012). Infectious disease emergence and global change: thinking systemically in a shrinking world. Infectious Diseases of Poverty.

[51] Daggy R, Page R (1956). Aramco’s preventive medicine program. Medical Bulletin.

[52] Franz KH (1968). Contributions of American industry in tropical medicine. Bull N Y Acad Med.

[53] Keiser J, Singer BH, Utzinger J (2005). Reducing the burden of malaria in different eco-epidemiological settings with environmental management: a systematic review. Lancet Infect Dis.

[54] Coffeng LE, Stolk WA, Zouré HGM, Veerman JL, Agblewonu KB, Murdoch ME, Noma M, Fobi G, Richardus JH, Bundy DAP, Habbema D, de Vlas SJ, Amazigo UV (2013). African programme for onchocerciasis control 1995–2015: Model-estimated health impact and cost. PLoS Negl Trop Dis.

[55] WHO (2012). Global report for research on infectious diseases of poverty. Special Programme for Research and Training in Tropical Diseases.

[56] Kleinschmidt I, Sharp B, Benavente LE, Schwabe C, Torrez M, Kuklinski J, Morris N, Raman J, Carter J (2006). Reduction in infection with Plasmodium falciparum one year after the introduction of malaria control interventions on Bioko Island, Equatorial Guinea. Am J Trop Med Hyg.

[57] Coyne PE, Berk DW (2001). The Mectizan (Ivermectin) Donation Program for Riverblindness as a Paradigm for Pharmaceutical Industry Donation Programs.

[58] Vagelos PR, Galambos L (2006). The Moral Corporation: Merck Experiences.

[59] World Economic Forum (WEF) (2004). Global Health Initiative: Private Sector Intervention Case Example: Using a Direct Service Model to Provide Workplace Prevention, Care, Support and Treatment.

[60] Krieger GR, Magnus M, Hassig S (2004). HIV/AIDS prevention programs: methodologies and insights from the dynamic modeling literature. Clinics in Occupational and Environmental Medicine.

[61] Fielding KL, Grant AD, Hayes RJ, Chaisson RE, Corbett EL, Churchyard GJ (2011). Thibela TB: design and methods of a cluster randomised trial of the effect of community-wide isoniazid preventive therapy on tuberculosis amongst gold miners in South Africa. Contemporary clinical trials.

[62] McBride D (2002). Missions for Science: U.S. Technology and Medicine in America’s African World.

[63] Mayson DT-W, Sawyer A (1979). Labour in Liberia. Rev Afr Polit Econ.

[64] United Nations (2006). Human Rights in Liberia’s Rubber Plantations: Tapping into the Future.

[65] Schechter PA (2012). Exploring the Decolonial Imaginary: Four Transnational Lives.

[66] Carter KR (2007). Amending the alien tort claims act: protecting human rights or closing off corporate accountability. Case Western Reserve JIL.

[67] Portney PR, Stavins RN (2000). Public Policies for Environmental Protection.

[68] Bhatia R, Wernham A (2008). Integrating human health into environmental impact assessment: an unrealized opportunity for environmental health and justice. Environ Health Perspect.

[69] Warhurst A (1998). Corporate social responsibility in the mining industries. MERN Research Bulletin and Newsletter.

[70] Maine D, Yamin AE (1999). Maternal mortality as a human rights issue: measuring compliance with international treaty obligations. HRQ.

[71] Clapham A, Rubio MG (2002). The obligations of states with regard to non-state actors in the context of the right to health. Health and Human Rights Working Paper Series.

[72] Salcito K, Utzinger J, Weiss MG, Münch AK, Singer BH, Krieger GR, Wielga M (2013). Assessing human rights impacts in corporate development projects. Environ Impact Assess Rev.

[73] Steinmann P, Keiser J, Bos R, Tanner M, Utzinger J (2006). Schistosomiasis and water resources development: systematic review, meta-analysis, and estimates of people at risk. Lancet Infect Dis.

[74] Al-Tuhami HA, Turkistani AM, Nooh RM (2001). Chickenpox outbreak among laborers in a company compound north of Riyadh, 2001. Saudia Arabia Saudi Epidemiology Bulletin.

[75] Cortes H, Morillas-Márquez F, Valero A (2003). Malaria in Mauritania: the first cases of malaria endemic to Nouakchott. Tropical Med Int Health.

[76] Jochelson K, Mothibeli M, Leger JP (1991). Human immunodeficiency virus and migrant labor in South Africa. International journal of health services: planning, administration, evaluation.

[77] Balfour-Kaipa TM (2012). Our journey on TB, HIV/AIDS in the South African mining industry.

[78] Schneider V (2014). Silicosis: The Curse of Lesotho’s Miners.

[79] Peipins LA, Lewin M, Campolucci S, Lybarger JA, Miller A, Middleton D, Weis C, Spence M, Black B, Kapil V (2003). Radiographic abnormalities and exposure to asbestos-contaminated vermiculite in the community of Libby, Montana, USA. Environ Health Perspect.

[80] Packard RM (1989). White Plague, Black Labor: Tuberculosis and the Political Economy of Health and Disease in South Africa.

[81] Jones BA, Grace D, Kock R, Alonso S, Rushton J, Said MY, McKeever D, Mutua F, Young J, McDermott J, Pfeiffer DU (2013). Zoonosis emergence linked to agricultural intensification and environmental change. PNAS.

[82] Benfica RMS (2006). An Analysis of Income Poverty Effects in Cash Cropping Economies in Rural Mozambique: Blending Econometric and Economy-Wide Models.

[83] Lecours N, Almeida GEG, Abdallah JM, Novotny TE (2012). Environmental health impacts of tobacco farming: a review of the literature. Tob Control.

[84] Guhl F, Pinto N, Aguilera G (2009). Sylvatic triatominae: a new challenge in vector control transmission. Mem Inst Oswaldo Cruz.

[85] Millen JV, Holtz TH, Kim JY, Millen JV, Irwin A, Gershman J (2000). Dying for growth, Part I: Transnational corporations and the health of the poor. Dying for Growth: Global Inequality and the Health of the Poor.

[86] Monshipouri M, Welch CEJ, Kennedy ET (2003). Multinational corporations and the ethics of global responsibility: Problems and possibilities. HRQ.

[87] Forsythe DP (2012). Human Rights in International Relations.

[88] Salama P, Laurence B, Nolan ML (1999). Health and human rights in contemporary humanitarian crises: is Kosovo more important than Sierra Leone?. BMJ.

[89] Feerick JD (2013). Doing well by doing good? A new normative perspective on corporate social responsibility. Fordham Environmental Law Review.

[90] Stiglitz JE (2003). Globalization and its Discontents.

[91] Meier BM, Fox A (2008). Development as health: employing the collective right to development to achieve the goals of the individual right to health. Human Rights Quarterly.

[92] Bernstein A, Greenwald C (2009). Benchmarking corporate policies on labor and human rights in global supply chains. Pensions and Capital Stewardship Project, Labor and Worklife Program.

[93] Kozarsky P, Keystone JS, Freedman DO, Nothdurft HD, Connor BA (2008). Travel Medicine E- Book: Expert Consult.

[94] Bayer R, Fairchild AL (2006). Changing the paradigm for HIV testing–the end of exceptionalism. N Engl J Med.

[95] King M, King E (2007). AIDS, Surgery and Life: A Malawi Mosaic.

[96] Baschetti R (2003). HIV/AIDS and human rights. Lancet.

[97] Corburn J (2004). Confronting the challenges in reconnecting urban planning and public health. Am J Public Health.

[98] Sexton K (2000). Socioeconomic and racial disparities in environmental health: is risk assessment part of the problem or part of the solution. Hum Ecol Risk Assess.

[99] Fox AM, Meier BM (2009). Health as freedom: addressing social determinants of global health inequities through the human right to development. Bioethics.

[100] Jobin W (1999). Dams and Disease: Ecological Design and Health Impacts of Large Dams, Canals and Irrigation Systems.

[101] Post Courier (2014). Stakeholders join hands to address health issues. Papua New Guinea Post Courier.

[102] Songco JA (2002). Do Rural Infrastructure Investments Benefit the Poor?: Evaluating Linkages: a Global View, a Focus on Vietnam.

[103] OECD (1996). Morocco - Socioeconomic influence of rural roads. Highway Project.

[104] Bates SJ, Trostle J, Cevallos WT, Hubbard A, Eisenberg JNS (2007). Relating diarrheal disease to social networks and the geographic configuration of communities in rural Ecuador. Am J Epidemiol.

[105] Magnussen L, Ehiri J, Jolly P (2004). Comprehensive versus selective primary health care: lessons for global health policy. Health Aff (Millwood).

[106] Kolbe-Alexander TL, Proper KI, Lambert EV, van Wier MF, Pillay JD, Nossel C, Adonis L, Van Mechelen W (2012). Working on wellness (WOW): a worksite health promotion intervention programme. BMC Public Health.

[107] Kolbe-Alexander TL, Conradie J, Lambert EV (2013). Clustering of risk factors for non-communicable disease and healthcare expenditure in employees with private health insurance presenting for health risk appraisal: a cross-sectional study. BMC Public Health.

[108] Zinsstag J, Schelling E, Bonfoh B, Fooks AR, Kasymbekov J, Waltner-Toews D, Tanner M (2009). Towards a ‘One Health’ research and application tool box. Vet Ital.

[109] Remais JV, Zeng G, Li G, Tian L, Engelgau MM (2013). Convergence of non-communicable and infectious diseases in low- and middle-income countries. Int J Epidemiol.

[110] Abebe SM, Berhane Y, Worku A, Assefa A (2014). Diabetes mellitus in North West Ethiopia: a community based study. BMC Public Health.

[111] Unwin N, Alberti KG (2006). Chronic non-communicable diseases. Ann Trop Med Parasitol.

[112] Krieger N, Birn AE (1998). A vision of social justice as the foundation of public health: commemorating 150 years of the spirit of 1848. Am J Public Health.

[113] Szreter S (1999). Rapid economic growth and ‘the four Ds’ of disruption, deprivation, disease and death: public health lessons from nineteenth-century Britain for twenty-first-century China?. Tropical medicine & international health: TM & IH.

[114] Brijnath B, Butler CD, McMichael AJ (2014). In an interconnected world: joint research priorities for the environment, agriculture and infectious disease. Infect Dis Poverty.

[115] Castro MC, Fisher MG (2012). Is malaria illness among young children a cause or a consequence of low socioeconomic status? evidence from the united Republic of Tanzania. Malar J.

[116] Paladin (2009). Paladin Energy Ltd - Investor Relations.

[117] Molyneux DH, Hotez PJ, Fenwick A (2005). “Rapid-impact interventions”: How a policy of integrated control for Africa’s neglected tropical diseases could benefit the poor. PLoS Med.

[118] WHO (2012). Technical report of the TDR disease reference group on zoonoses and marginalized infectious diseases of poverty. WHO Tech Rep Ser.

[119] Zinsstag J, Schelling E, Wyss K, Mahamat MB (2005). Potential of cooperation between human and animal health to strengthen health systems. Lancet.

