# Supplementary material for: Multinational corporations and infectious disease: Embracing human rights management techniques
Source: Infect Dis Poverty. 2014 Nov 3;3:39. doi: 10.1186/2049-9957-3-39 (PMC4323175; doi:10.1186/2049-9957-3-39)
Supplement: Supplementary file 1 — Additional file 1:Multilingual abstracts in the six official working languages of the United Nations.(PDF 330 KB) [file 40249_2014_80_MOESM1_ESM.pdf]

Translation of the abstract into the six official working languages of the United Nations

الشركات المتعددة الجنسيات والأمراض المعدية: تبني أساليب إدارة حقوق الإنسان

كيندل سالسيتو، بيرتون هـ سنجر، ميتشيل ج ويس، ميركو س وينكلر، جاري ر كريجر، مارك ويلجا و جورج يوتزنجر

الملخص

الخلفية: دعت المؤسسات الصحية العالمية الحكومات، والمنظمات الدولية والممارسين الصحيين لتوظيف نهج قائم على حقوق الإنسان للأمراض المعدية. الدافع لنهج حقوق الإنسان واضح: الفقر وعدم المساواة يهيئان الظروف للأمراض المعدية للانتشار، والأمراض، بدورها، تتفاعل مع النظم الاجتماعية البيئية لنشر الفقر، وعدم المساواة والإهانة. يجب أن تهتم الحكومات والمنظمات الحكومية الدولية بالسيطرة والقضاء على هذه الأمراض، لأن انتشار العدوى يؤخر النمو الاقتصادي ويساهم في ارتفاع تكاليف الرعاية الصحية وبطء العمل لتحقيق حقوق الإنسان الشاملة. ويجب لهذه المحددات الاجتماعية والنتائج الاقتصادية المرتبطة بالأمراض المعدية أن تثير اهتمام الشركات متعددة الجنسيات، ويرجع ذلك إلى حد ما لتأثيرها على إنتاجية الشركات، وبشكل متزايد، لأن المبادئ العالمية الجديدة تفرض على الشركات مسؤولية احترام حقوق الإنسان، بما فيها الحق في الصحة.

طرق العمل: استعرضنا التطورات التاريخية والحديثة في تفاعل الأمراض المعدية، وحقوق الإنسان والشركات متعددة الجنسيات. وتم استكمال تحقيقنا مع رؤى على المستوى الميداني في المشروعات الرأسمالية للشركات التي تم تطويرها في مناطق توطن عال للأمراض المعدية، التي تبنت استراتيجيات لمكافحة المرض قائمة على الحقوق.

النتائج: الخبرة والمواد المطبوعة توفر حالة عمل ممتدة وحالة ناشئة من المسئولية الاجتماعية للشركات لتطبيق نهج حقوق الإنسان في برامج الصحة بفعاليات شاملة. بالفعل، في عالم تزداد فيه العولمة والترابط، نجد أن الشركات متعددة الجنسيات لديها مصلحة، ودور مهم تقوم به، في تعزيز استراتيجيات مكافحة الأمراض المعدية القائمة على الحقوق.

الاستنتاج: هناك فرص جديدة للحكومات والهيئات الصحية الدولية لحشد الأطراف الفاعلة من الشركات التجارية في استراتيجيات المكافحة والقضاء على الأمراض. الإرشادات التي قدمتها الأمم المتحدة في عام 2011 والتي احتضنت على نطاق واسع من قبل الشركات والحكومات والمجتمع المدني تشكل خريطة طريق لإشراك المؤسسات التجارية في استراتيجيات إدارة الأمراض القائمة على الحقوق للتخفيف من معدلات انتقال المرض وتحسين نتائج رفاهية الإنسان.

Translated from English version into Arabic by Saher Salama, through

TRANSLATORS
WITHOUT BORDERS

跨国公司与传染病：应用人权管理技术

Kendyl Salcito, Burton H Singer, Mitchell G Weiss, Mirko S Winkler, Gary R Krieger, Mark Wielga and Jürg Utzinger

摘要

引言： 全球卫生机构已要求政府、国际组织和卫生工作者应用一种基于人权的措施应对传染病。这种方法的动机清晰：贫穷和不平等为传染病繁衍创造了条件，反过来，传染病影响社会生态系统，进一步导致贫穷、不平等和侮辱。政府和国际组织应关注这些疾病的控制和消除，因为普遍的传染病会阻碍经济增长，导致更高的医疗成本，推迟实现人权的进程。这些与传染病有关的社会决定因素和经济产出应该引起跨国公司的关注，部分是因为它们与公司生产力有关，更多的是因为新的全球规范要求公司要尊重人权，这包括健康权。

方法： 回顾传染病、人权和跨国公司相互关系的历史和最近发展，并补充了一些在传染病高流行区开展包括基于人权的疾病控制策略在内的公司资本项目发展的现场见解。

结果： 现场经历和文献为公司提供了一个长期存在的商业案例和一个新兴的社会责任案例，使企业在全球健康业务中应用基于人权的措施。在日益全球化和相互联系的社会，跨国公司在促进基于人权的传染病控制策略方面确实具有重要的作用。

结论： 政府和国际卫生机构拥有新的机遇得到公司在疾病控制和消除方面的资助。2011 年联合国提供的且受到公司、政府和民间团体广泛欢迎的指导手册为商业企业从事基于人权的疾病管理策略从而缓解疾病传播率和促进人类福祉提供了发展蓝图。

Translated from English version into Chinese by Yin Jian-hai, through

CDC
CHINESE CENTER FOR DISEASE CONTROL AND PREVENTION
NATIONAL INSTITUTE OF PARASITIC DISEASES

Sociétés multinationales et maladies infectieuses: adhésion aux techniques de gestions des Droits de l'Homme

Kendyl Salcito, Burton H Singer, Mitchell G Weiss, Mirko S Winkler, Gary R Krieger, Mark Wielga and Jürg Utzinger

Sommaire

Contexte: les institutions mondiales de la santé ont appelé les gouvernements, les organismes internationaux et les professionnels de santé à adopter une approche basée sur les droits de l'homme, en ce qui concerne les maladies infectieuses. La motivation pour une approche des droits de l'homme est claire: la pauvreté et les inégalités créent les conditions pour que les maladies infectieuses se développent. A leur tour, ces maladies ont une interaction sur les systèmes socio-écologiques pour générer la pauvreté, l'injustice et l'indignité. Les gouvernements et les organismes intergouvernementaux devraient se préoccuper de contrôler et d'éliminer ces maladies, car la propagation de ces infections freine la croissance économique, génère des frais de santé plus élevés et ralentit les processus de réalisation universelle des droits de l'homme. Ces déterminants sociaux et ces résultats économiques associés aux maladies infectieuses devraient interpeler les sociétés multinationales, en partie parce qu'elles ont une influence sur la productivité des entreprises et, de plus en plus, parce que les nouvelles normes mondiales imposent aux sociétés la responsabilité de respecter les droits de l'homme, y compris le droit à la santé.

Méthodes: Nous avons revu les récents développements historiques à l'interface des maladies infectieuses, des droits de l'homme et des sociétés multinationales. Notre enquête a été complétée par un aperçu, au niveau du terrain, des projets d'immobilisations qui ont été développés dans des régions fortement endémiques de maladies infectieuses qui ont adhéré aux stratégies de contrôle des maladies basées sur les droits de l'homme.

Résultats: L'expérience et la littérature fournissent une longue analyse de rentabilisation et une responsabilité sociale nouvelle pour que les sociétés adoptent une vision basée sur les droits de l'homme dans les programmes de santé des opérations mondiales. En effet, dans un monde de plus en plus globalisé et interconnecté, les sociétés multinationales ont un intérêt, et un important rôle à jouer, pour promouvoir des stratégies de contrôle liées aux droits de l'homme en ce qui concerne les maladies infectieuses.

Conclusions: Il y a de nouvelles opportunités pour que les gouvernements et les organismes de santé internationaux mobilisent les acteurs des entreprises dans des stratégies de contrôle et d'élimination des maladies. Des conseils prodigués par les Nations Unies en 2011 - et qui sont largement suivis par les sociétés, les gouvernements et les sociétés civiles - fournissent une feuille de route pour l'engagement des entreprises dans des stratégies de gestion des maladies basées sur les droits de l'homme, afin de réduire les taux de transmission des maladies et d'améliorer les résultats du bien-être humain.

Translated from English version into French by Ode Laforge, through

TRANSLATORS
WITHOUT BORDERS

Транснациональные корпорации и инфекционные заболевания: внедрение методов управления, направленных на соблюдение прав человека

Кендил Салсито, Бартон Х. Сингер, Митчелл Г. Вайс, Мирко С. Винклер, Гари Р. Кригер, Марк Виелга и Юрг Утцингер

Резюме

История вопроса: Всемирные учреждения по охране здоровья призвали правительства, международные организации и медицинский персонал применять подход, основанный на соблюдении прав человека, к людям, страдающим инфекционными заболеваниями. Мотивы этого призыва понятны: бедность и неравенство создают предпосылки для распространения инфекционных заболеваний, и эти заболевания, в свою очередь, в сочетании с социально-экологическими системами, ведут к бедности, неравенству и недостойным условиям жизни. Правительства и межправительственные организации должны стремиться к контролю и ликвидации этих заболеваний. Широкое распространение инфекций замедляет рост экономики и вызывает рост расходов на здравоохранение, а также замедляет прогресс соблюдения всеобщих прав человека. Социальные детерминанты и экономические последствия, связанные с распространением инфекционных заболеваний, должны интересовать транснациональные корпорации, отчасти потому, что они влияют на их продуктивность и, в большей степени, потому, что новые всеобщие нормы поведения налагают на эти компании ответственность за соблюдение прав человека, включая право на здоровье.

Методы: мы рассмотрели исторические и новейшие тенденции взаимосвязей между инфекционными заболеваниями, правами человека и деятельностью транснациональных корпораций. Наше исследование было дополнено аналитическими выводами по инвестиционным проектам корпораций на местном уровне, разработанными в зонах высокой эндемичности инфекционных заболеваний, в соответствии со стратегиями контроля заболеваний, основанными на соблюдении прав человека.

Результаты: опыт и литература по данному вопросу доказывают долгосрочную экономическую выгоду и возрастающую социальную ответственность корпораций за соблюдение прав человека в процессе создания программ по здравоохранению в глобальных масштабах. Безусловно, в условиях нарастающей глобализации и всё более взаимосвязанного мира, усовершенствование стратегий контроля заболеваний, основанных на соблюдении прав человека, выгодно транснациональным корпорациям, которые должны играть важную роль в этом процессе.

Выводы: существуют новые возможности для правительств и международных здравоохранительных организаций получить поддержку от участников корпоративного бизнеса в разработке стратегий контроля и искоренения этих заболеваний. Рекомендации, предложенные ООН в 2011 году, широко используемые компаниями, правительствами и общественностью, являются путеводной нитью для предприятий, занимающихся бизнесом. Они способствуют разработке стратегий лечения заболеваний, основанных на соблюдении прав человека, снижению скорости распространения заболеваний и повышению благосостояния населения.

Translated from English version into Russian by Maria Sitnikova, through

TRANSLATORS
WITHOUT BORDERS

Multinacionales y enfermedades infecciosas: adoptando técnicas de gestión de derechos humanos

Kendyl Salcito, Burton H Singer, Mitchell G Weiss, Mirko S Winkler, Gary R Krieger, Mark Wielga and Jürg Utzinger

Resumen

Antecedentes: Las instituciones sanitarias globales exigen a los gobiernos, organizaciones internacionales y médicos que traten las enfermedades infecciosas desde el punto de vista de los derechos humanos. El motivo de dicho enfoque es evidente: la pobreza y la desigualdad crean las condiciones para la proliferación de las enfermedades infecciosas, y éstas a su vez, interactúan con los sistemas socio ecológicos para difundir pobreza, desigualdad y humillación. Los gobiernos y organizaciones intergubernamentales deberían preocuparse por el control y la eliminación de estas enfermedades, ya que las infecciones generalizadas ralentizan el crecimiento económico y contribuyen al aumento de los gastos sanitarios y procesos más lentos de desarrollo de derechos humanos universales. Estos determinantes sociales y consecuencias económicas asociados a enfermedades infecciosas deberían interesar a las multinacionales, en parte porque repercuten en la productividad empresarial y cada vez más porque las normas globales imponen sobre las empresas la responsabilidad de respetar los derechos humanos, incluido el derecho a la salud.

Métodos: Hemos revisado los desarrollos históricos y recientes en la interrelación de enfermedades infecciosas, derechos humanos y multinacionales. Nuestra investigación se complementó con perspectivas a nivel de terreno en proyectos de capital social llevados a cabo en zonas altamente endémicas de enfermedades infecciosas, que adoptaron estrategias de control basadas en los derechos humanos.

Resultados: La experiencia y la literatura facilitaron un business case de muchos años y un caso de responsabilidad social incipiente para que las empresas apliquen un enfoque de derechos humanos a los programas de salud en operaciones globales. De hecho, en un mundo cada vez más globalizado e interconectado, a las multinacionales les interesa, y tienen un importante papel a desempeñar en estrategias avanzadas de control de enfermedades infecciosas basadas en los derechos humanos.

Conclusión: Existen nuevas oportunidades para Gobiernos e instituciones sanitarias internacionales de reclutar actores empresariales para estrategias de control y eliminación de enfermedades. La orientación ofrecida por las Naciones Unidas en 2011, ampliamente adoptada por empresas, gobiernos y sociedad civil, proporciona una hoja de ruta para comprometer a las empresas en la gestión de enfermedades basada en los derechos humanos, con el fin de mitigar los índices de transmisión y mejorar los resultados del bienestar humano.

Translated from English version into Spanish by Raquel Bentué, through

TRANSLATORS
WITHOUT BORDERS
